# Leprosy

**Published:** 1894-05

**Authors:** Isadore Dyer

**Affiliations:** New Orleans, La., Professor of Dermatology in the New Orleans Polyclinic; Lecturer and Clinical Instructor in Skin Diseases, Medical Department Tulane University, etc.


For Texas Medical Journal.

### LEPROSY.

BY ISADORE DYER, M. D., NEW ORLEANS, LA.,
Professor of Dermatology in the New Orleans Polyclinic; Lecturer and Clinical Instructor in Skin Diseases, Medical Department Tulane University, etc.

LEPROSY is an endemic, malignant, constitutional disease, characterized by alterations of the cutaneous, nerve and bone structures, resulting in anæsthesia, ulceration, necrosis, general atrophy, and deformity. Leprosy is a well defined affection, due to the development in the economy of a special bacillus, called the bacillus lepræ, or the bacillus of Hansen. This

---

Lecture delivered to the medical class, 1894.

bacillus is rod-shaped, with conical ends, or rounded, resembling the bacillus of tuberculosis, but shorter, pointed, and more of a cylinder than the latter. The bacillus of Hansen is very tenacious of life, and resists to a remarkable degree the action of time and atmospheric agents. In its development in the tissues, the micro-organism causes the formation of neoplasms, or new growths. These neoplasms formed in the skin or the mucous membranes, give rise to the tubercular type, while, attacking the nerves especially, they develop the anæsthetic, or the typhoneurotic variety of leprosy. When these two forms are intercurrent, the type then present is called the "mixed," or "complete" variety. Often the one, especially the tubercular type, disappears, giving place to the other as a sequel. Another form of leprosy is sometimes described, the macular. This seems to be only a stage in the development of either of those already mentioned.

Leprosy is ushered in by certain prodromic manifestations, to which has been given the name, period of invasion. This stage of the disease is marked by fevers, irregular in period and in type. Malaise, anorexia, dyspepsia, epistaxis, dryness of the nasal passages and of the respiratory tract, vertigo, headaches, exaggeration of the functions of the fat glands, are some of the notable symptoms. There may be pruritus or hyperæsthesia of the skin, and neuralgic pains in all locations. There is often a premonitory eruption, of the bullous type, resembling pemphigus. It differs, however, in this, the eruption seems to affect the extremities chiefly, comes in rapidly successive crops of vesicles, or bullæ, which, breaking, often heal as ulcers. As introductory, now the macular eruption appears. At various parts of the body, spots appear, red at first, then brownish; the borders become white, and occasionally thickened. These fade, often shortly after their appearance; at times, some are fading while others are developing. After a lapse of time, months, or years even, the symptoms of confirmed leprosy appear. These may be characteristic at the start. At once leprosy may appear in small tubercules. Oftenest, however, the first eruption to appear consists of spots, or patches, varying in size, but averaging the size of the palm. These are hyperæmic or erythematous, a pale red or wine color, at times violaceous, livid or brown, yellow, or even plainly pigmented, or almost black.

These spots sometimes disappear completely, even quite rapidly, and without leaving any traces. The center is sometimes darker than the margin, sometimes depressed and free from color, the patch being formed by the ring of its periphery. It is frequent, at this stage, to see nodosities scattered over the skin, resembling the nodular erythema, accompanied likewise by rise of temperature, and being transitory and leaving abruptly at times. All such symptoms may be absent, and the lepra tubercle be evident from the beginning. This is a sort of rounded, hemispherical nodosity, varying in size from a pin's head to a hazel nut, hard and elastic to the touch, pale red or brown in color, at times copper colored, smooth and telangiectic. These tumors are isolated and form distinct, discrete nodosities, or they may be confluent, forming irregular shaped, rounded, oblong masses. They may develop on the patches referred to above. In that case, the patches become thickened at certain points, and here the nodosities are formed.

Most frequently the tubercules occur in the corium, then, secondarily, they invade the adjacent tissues. They may be entirely underneath the skin, but though not seen, they can be readily felt. The most frequent locations of this, the tubercular, type of leprosy, are the face, the hands, the forearms, and the lower limbs. Attacking the face, the forehead, eyelids, nose, lips, chin and cheeks suffer most. The nose is flattened, enlarged and infiltrated. The cheeks are bunched. The ears, lobules particularly, are thickened, pedunculated, and leathery; tubercles as large as hazel nuts often develop here. The scalp is rarely affected. Once developed, the tubercles do not remain stationary. They may continue to grow, become confluent, form enormous bunches; they may exfoliate, or become complicated with œdema. Spontaneous retrogression sometimes occurs. The lesions soften, grow pale, sink into the skin, shrivel up, and finally disappear, leaving behind a spot with a yellow-white center and a pigmented periphery. They may become inflamed, suppurate, open on the surface, and slough in part or entire. They may simply ulcerate, without destruction of tissue, remain small, superficial ulcers, covered with greenish or brown crusts, destroying, by degrees, the adjacent tissues underneath, the tendons, ligaments, and the bones.

Tubercles developing on the mucous membranes tend to ulcerate and produce destruction or functional disturbances of the parts involved. From the beginning, there are disturbances of the various senses. In certain cases, the tubercular eruption develops very rapidly, and may reach a fatal termination in a few months; more often, however, it takes years to complete its course. There are periods of latency and activity, but death comes finally, from exhaustion, from marasmus, or is hastened by some intercurrent malady, of which tuberculosis, syphilis and nephritis, are the most frequent.

The anæsthetic, or the tropho-neurotic form of leprosy, or, as Leloir calls it, the systematic nervous leprosy, presents the same period of invasion as the tubercular form. The bullous eruption usually appears in single lesions, which are incessantly reformed, leaving behind a scar, or at least a pigmentation. Here, too, the bullæ may come on the ends of the fingers or toes, and leave very obstinate sores. Usually this form comes as a skin eruption, erythematous or hyperæmic at first, then colored or not. It may be pigmented at the start, with subsequent atrophy of the pigment in whole or in part. The spots of most importance, and usually looked upon as characteristic of the macular stage of anæsthetic leprosy, are patches, smooth and shining, with well defined periphery, free from color, atrophied in the center, resembling somewhat the lesions of morphœa and vitiligo. The edges are colored red, brown, or brownish yellow, and may or may not be prominent or elevated. These patches are often serpiginous, but in such cases the result of confluence. These patches are the seat of frequent disturbances of sensation. The discolored parts are always anæsthetic, and those most colored are the most anæsthetic. In exceptional cases, the reverse is the case; namely, instead of anæsthesia, these patches are markedly hyperæsthetic. The anæsthesia of this form of leprosy may occur at points free from patches or spots, or apparently free from a lesion. In such event, the seat of the anæsthesia is on the forgotten site of an injury, bruise, or burn.

Gradually the nervous system is involved. Leloir speaks of two divisions of the leprous nerve effect (Traite de la lepre, 1886): 1. The period of invasion. This corresponds to the period of the cutaneous manifestations, when hyperæsthesia is common, when paroxysms of neuralgic pains occur, rheumatic, or arthritic pains, and it is possible to observe marked thickening of certain nerves.

2. A period of nerve degeneration, marked clinically by anæsthesia, paralyses, atrophies, and trophic disturbances. With the anæsthesia, there appears a muscle atrophy, which attacks, first of all, the muscles of the hand (causing contraction), the extensor and flexor muscles of the forearm, and the characteristic

"*griffe*," or "claw hand," follows. There is loss of power, sense of touch, etc., in these parts. The muscles of the foot and leg are likewise similarly affected. At times, the muscles of the face and the trunk are affected. Then comes the atrophy of the skin, the shortening of the muscles, shrinking of the skin, and a general senile aspect. There are a variety of tropic disturbances, shedding of the nails, falling of the hair, loss of the teeth, ulceration of the nasal passages, and ulceration of the gums. There may be perforating ulcers of the feet and hands,—ulcers, painless, anæsthetic, beginning over the joints, gradually deepening, and ending by extending to the articulation, causing the phalanges of the fingers or the toes to fall. Dry gangrene necrosis, with abscesses, occur, absorption of bone, and a final deformity of the patient results. Now the tropho-neurotic leprosy has reached its last stage. Marasmus begins, with general listlessness, and the patient dies from pure exhaustion, or death is hastened by an almost necessary septicæmia. Often a complication with pneumonia, pleurisy, albuminuria, or a persistent diarrhœa, carries the patient off.

The mixed, or complete form of leprosy, is the really typical form. Here there is a combination of the tubercular and the anæsthetic varieties. It may begin as the mixed, or, starting as tubercular leprosy, it may assume the anæsthetic form, the two being intercurrent. It then, of course, assumes the symptoms of both varieties, and the history and the end is the same.

These three forms may so vary as to deceive, and a part only of the symptoms be present. In this, leprosy does not differ from other diseases, but if the mental photograph of a type is always ready, the disease is not hard to diagnose, even in its exceptions.

The bacillus of leprosy is contained often in the lymphatic cells and tissues, but is most frequently found in the leprous tissues themselves. Leprous tissue is granulation tissue, made up of spheroidal cells, massed so as to infiltrate the corium, and breaking up the connective tissue fibres. These cells are grouped around the vessels, these hypertrophy become varicose, and are themselves much thickened. Ultimately the whole or part of this leprous tissue is re-absorbed or eliminated, and the site is marked by a cicatrix.

From syphilis, leprosy is diagnosed by the color of the lesions, their course of development, the anæsthesia, the deformity, and finally the microscopic finding of the Hansen bacillus.

It is diagnosed from morphœa and circumscribed scleroderma, by the location, general distribution of the lesions, and the ulceration. Further the lesions of leprosy are usually anæsthetic, while those of these affections are exceptionally so.

Finally leprosy can be isolated from other affections if you are mindful of these points.

1. Habitat. 2. History of contact. 3. Anæsthesia. 4. Trophic disturbances. 5. Eruptions of bullæ in successive crops, or single one recurring. 6. Perforating ulcers. 7. Muscle atrophy. 8. The claw hand. 9. Clubbed fingers. 10. Discolored and blunted nails. 11. Characteristic anæsthesia of the little finger an early sign. 12. The leonine face. 13. The leathery ears. 14. Ectropion. 15. Deformity, and loss of phalanges of fingers and toes.

The cause of leprosy is the bacillus lepræ or the bacillus of Hansen, sometimes called the bacillus of Niessler. The disease is contagious, and by inoculation. The bacillus is brought in contact with a broken surface, accepted by the circulation, and is in time spread generally over the body. The disease may be congenital, but has not yet been proven hereditary.

There are numerous contributing causes, chief among which are poverty and bad hygienic conditions. Improper diet and exposure are also factors. It is probable that the disease is more common along the sea board than in the interior counties, but climatic conditions are only of secondary importance.

Many authors look upon fish diet as productive of the disease, but the disease has been found prevalent in sections where fish diet was not possible.

Leprosy may get well spontaneously. The disease, however, is generally considered incurable. The treatment of leprosy is tonic. A change of climate is advisable, and the plainest possible diet. Regular tonic baths, cold douches, showers, and alkaline. Numerous remedies have been suggested, but most of them are only palliative, excepting in a very few cases. Of the remedies used, Chaulmoogra oil is the most popular. It is given internally, beginning with five drops three times a day after eating, and gradually increasing. It is best given in capsule or in cold tea or in milk.

Hoang Nan is a remedy much used in South America. It is given in pill form in doses of three grains three times a day after meals. Arsenious acid, sulphate of strychnine in tonic doses, and long continued, are given. Salicylate of soda, quinine, salol, iodide and bromide of potassium are also given. Unna, of Hamburg, has succeeded in two cases with the use of ichthyol, internally and externally. Internally he gave the drug in increasing doses to a point of tolerance, beginning with five drops three times a day. Externally it was used in ointments and plasters, alone and combined with resorcin, with pyrogallic acid, etc.

The external treatment of leprosy should be based on two essential principles. First the removal of the lesions with caustics or cautery, and second, promotion of the absorption of the lesions with suitable appplications. For the tubercles, in their early stage, iodine, nitrate of silver, blisters, mercurial ointments, electro-cautery, etc., may be used.

Balsam of Peru ointment, iodide of lead ointment, salicylic acid ointment, gurgun oil, etc., may be rubbed into the lesions.

When there is ulceration, ordinary antiseptic methods should be applied. Iodoform, salol, boric acid, aristol, etc., may be dusted on, after thorough cleansing with antiseptic solutions.

Where the lesions of anæsthetic leprosy are conveniently confined to one member, it is advisable to stretch the principal nerve or even to make an exsection of the nerve.

The prognosis of leprosy is always bad. The disease may be arrested, temporarily relieved, but cures are rare. The disappearance of all evidences of the disease may be followed years later by a new manifestation and with manifold energy.

The tubercular is more rapidly fatal than the other form. According to Hillis (Ziemssen), 38 per cent. die of leprosy and its direct consequences. The rest of the fatal cases die of nephritis, pneumonia, diarrhœa, anemia, fevers, peritonitis,—in the order named.

In the prophylaxis of leprosy the patient should be made to take regular baths, and to use individual utensils, towels, etc. Excesses of all kinds should be avoided. An occupation should be followed which would not endanger family or friends.

For the protection of the public, lepers should be isolated, should be prohibited from marrying, even among themselves.

The disease is insidious, and the consensus of the best medical opinion favors absolute quarantine, with complete isolation, as the only means of entirely suppressing the disease.

A word as to the history: At the time of Christ, leprosy was still prevalent in the East. It existed, probably confined, in the early history of the world, to Egypt and the Orient. In the first century, invading Greece, it spread over Southern Europe. In the 11th and 12th centuries, during the Crusades, it spread all over Europe, reaching then the acme of its force, finally materially disappearing from the 15th to the 17th century. During this time, it was estimated that there were 19,000 lepers in Europe, 2000 of them in France alone. At the present time, the disease is endemic in Northern and Eastern Africa, Madagascar, Arabia, Persia, India, China, Japan, Liberia, and the islands of the Pacific and Indian oceans. In Europe, leprosy is still active in Norway, Southern Russia, points along the Mediterranean, and in Brittany, in France. In North America, it is found in Canada and in the United States. It is endemic in Louisiana, where it was introduced in 1758, by the Acadians. Various attemps have been made at colonization here, but with hardly enough spirit to assure success. This has been successfully accomplished in Minnesota, where leprosy occurs among the Norwegian settlers. In 1859, leprosy was introduced into the Sandwich Islands, by two Chinese immigrants. In 1885, there were 4500 cases in the Sandwich Islands. Leprosy has existed in Mexico since Cortez's time.

There seems in this country a certain apathy in the matter of the care of our lepers. With such an example as the Sandwich Islands afford, and in a climate far superior to our own, it seems a foolhardy indifference which exposes all to the common risk. Suitable legislation should be demanded and its enforcement compelled.

